# Supplementary material for: Genomic Surveillance and Molecular Evolution of Fungicide Resistance in European Populations of Wheat Powdery Mildew
Source: Mol Plant Pathol. 2025 Mar 19;26(3):e70071. doi: 10.1111/mpp.70071 (PMC11922816; doi:10.1111/mpp.70071)
Supplement: Supplementary file 1 — Figure S1. [file MPP-26-e70071-s004.pdf]

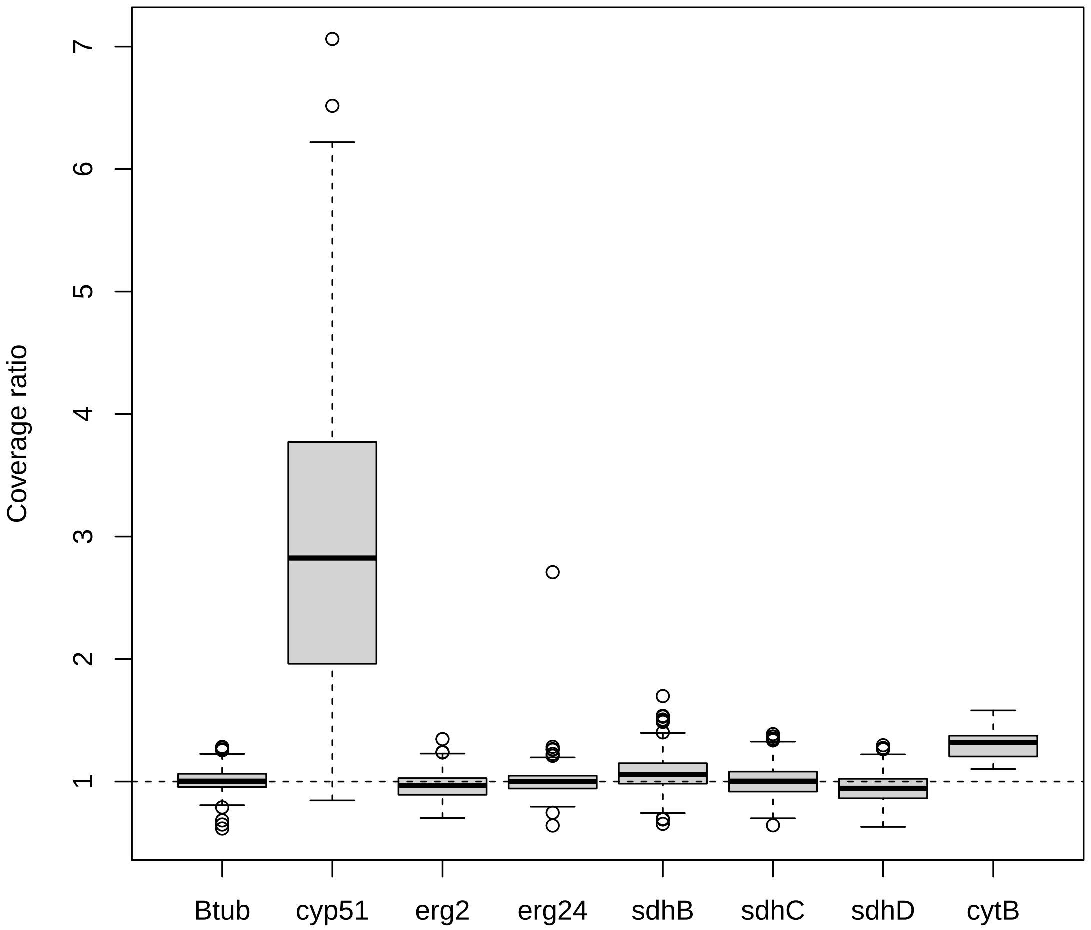


**Figure S1. Coverage ratio for the eight studied genes**
The ratios of gene coverage and genome-wide coverage were used to estimate the number of copies for each gene. For *cytb* we used the mitochondrial genome coverage as denominator.
