# Supplementary material for: Genomic Surveillance and Molecular Evolution of Fungicide Resistance in European Populations of Wheat Powdery Mildew
Source: Mol Plant Pathol. 2025 Mar 19;26(3):e70071. doi: 10.1111/mpp.70071 (PMC11922816; doi:10.1111/mpp.70071)
Supplement: Supplementary file 2 — Figure S2. [file MPP-26-e70071-s002.pdf]

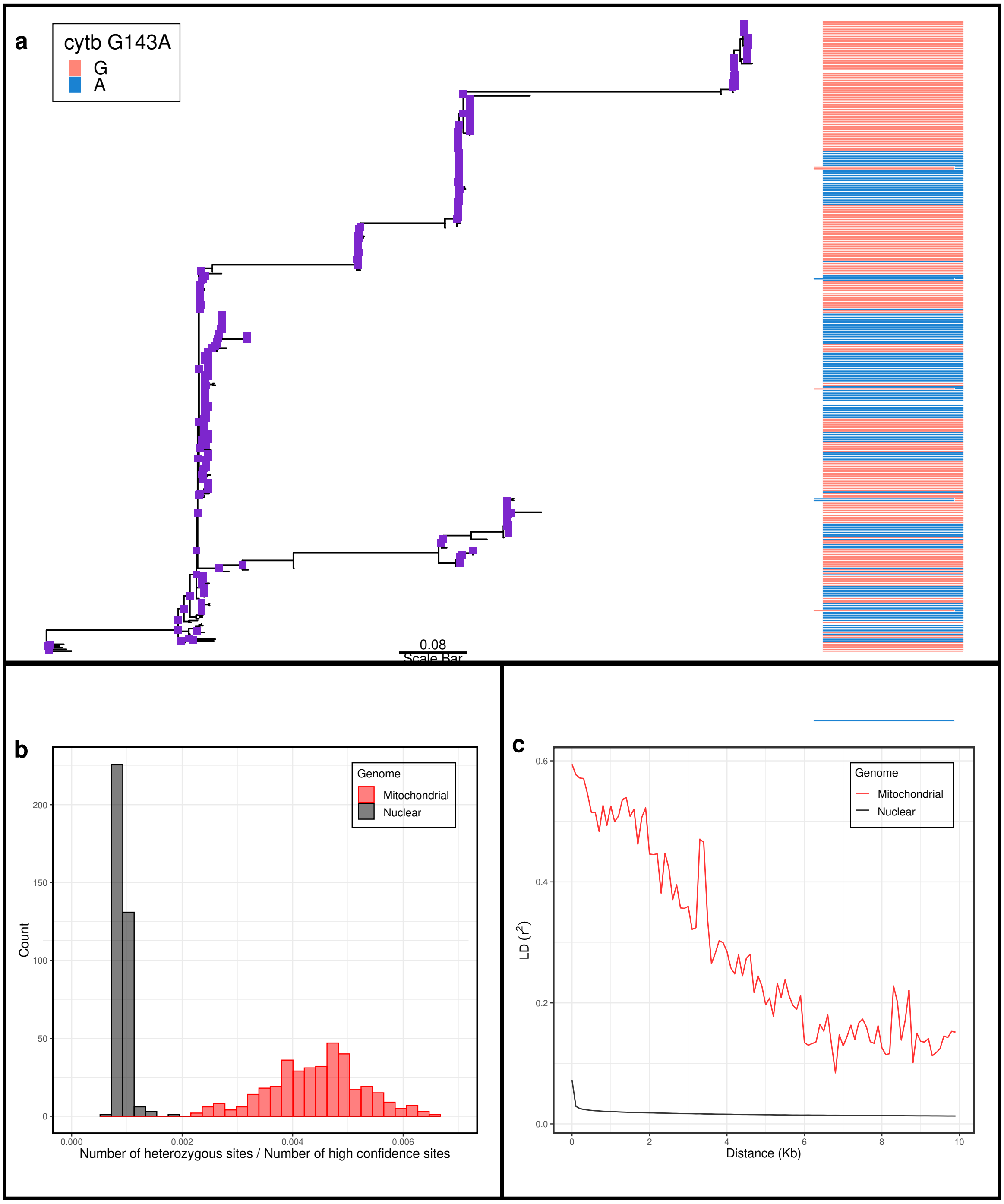

Figure S2. Evolution of *cytb* and the mitochondrial genome
(a) Mitochondrial phylogenetic tree for the *Europe+_recent* dataset rooted with five rye powdery mildew isolates. Purple squares represent nodes with less than 50% bootstrap support. The bars on the right indicate which amino acid is present at position 143 in *cytb*. **(b)** Frequency of heterozygous mutations in the nuclear and mitochondrial genome. **(c)** LD decay in the nuclear and mitochondrial genome.
