# Supplementary material for: Genomic Surveillance and Molecular Evolution of Fungicide Resistance in European Populations of Wheat Powdery Mildew
Source: Mol Plant Pathol. 2025 Mar 19;26(3):e70071. doi: 10.1111/mpp.70071 (PMC11922816; doi:10.1111/mpp.70071)
Supplement: Supplementary file 3 — Figure S3. [file MPP-26-e70071-s014.pdf]

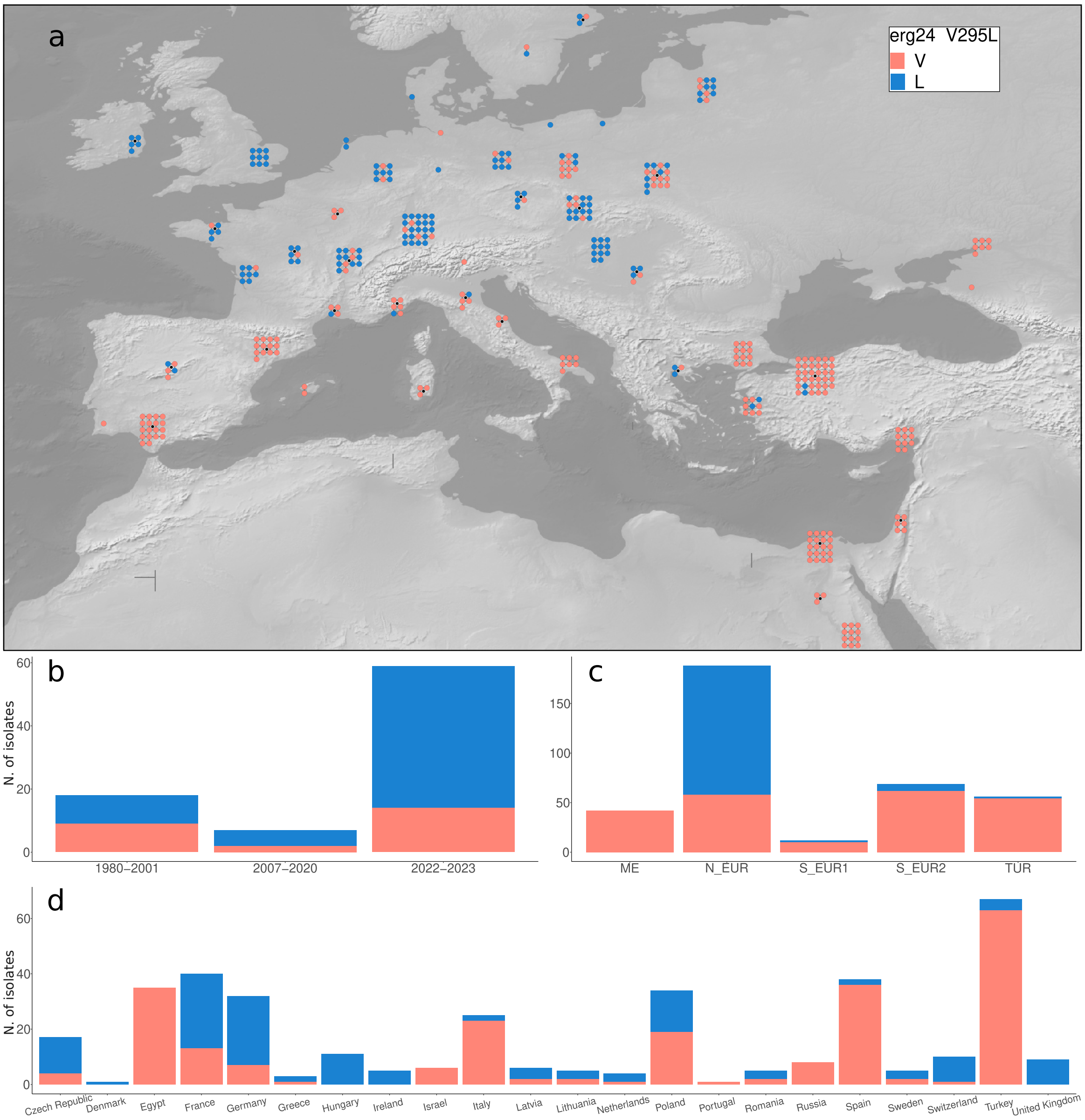

Figure S3. ***erg24*** **mutation V295L**
(a) Distribution of V295L. **(b)** Frequency of V295L by year of collection (*temporal* dataset). **(c)** Frequency of V295L by population. **(d)** Frequency of V295L by country of origin.
