# Supplementary material for: Genomic Surveillance and Molecular Evolution of Fungicide Resistance in European Populations of Wheat Powdery Mildew
Source: Mol Plant Pathol. 2025 Mar 19;26(3):e70071. doi: 10.1111/mpp.70071 (PMC11922816; doi:10.1111/mpp.70071)
Supplement: Supplementary file 4 — Figure S4. [file MPP-26-e70071-s017.pdf]

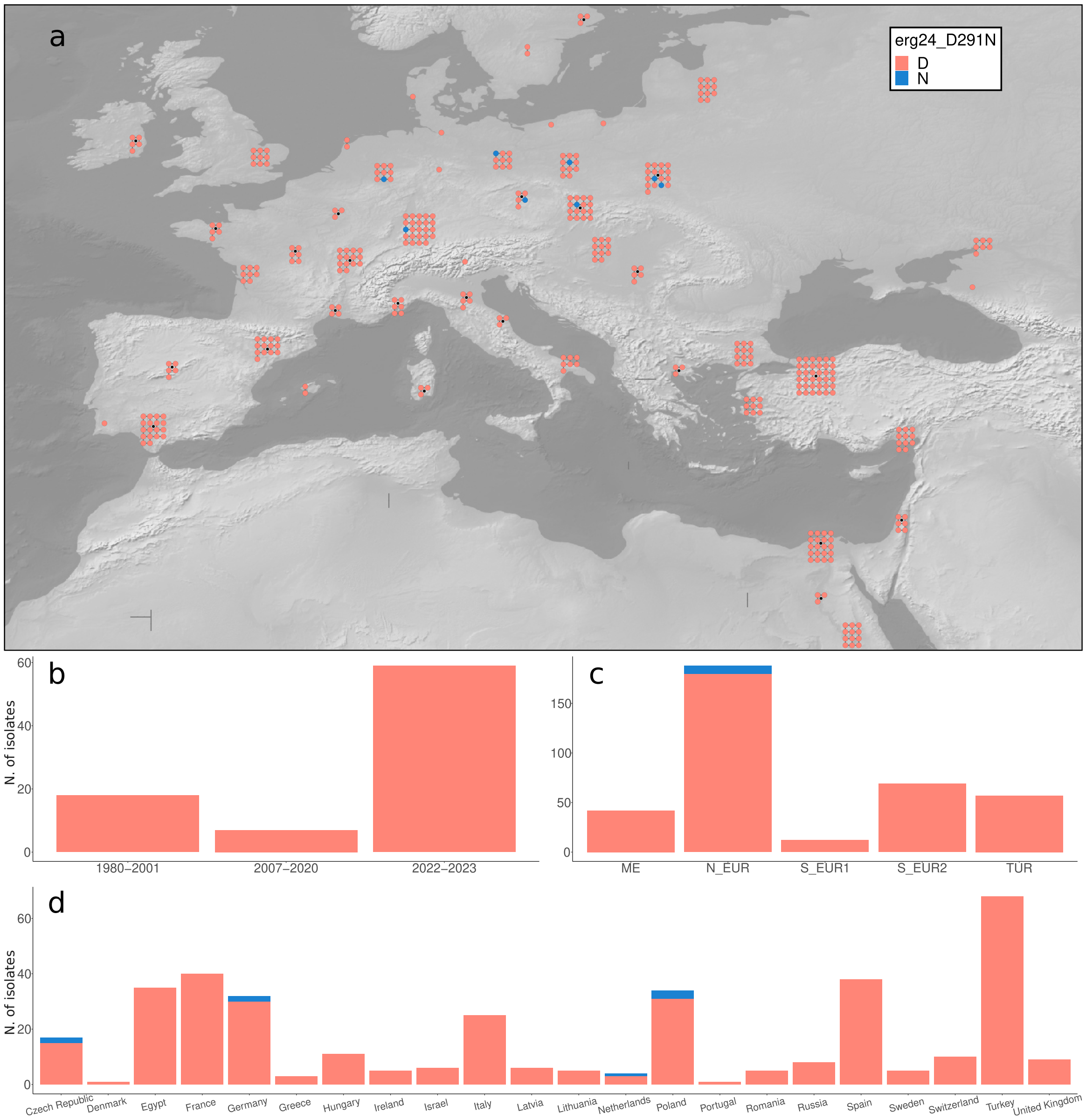

Figure S4. ***erg24*** **mutation D291N**

(a) Distribution of D291N. **(b)** Frequency of D291N by year of collection (*temporal* dataset). **(c)** Frequency of D291N by population. **(d)** Frequency of D291N by country of origin.
