# Supplementary material for: Genomic Surveillance and Molecular Evolution of Fungicide Resistance in European Populations of Wheat Powdery Mildew
Source: Mol Plant Pathol. 2025 Mar 19;26(3):e70071. doi: 10.1111/mpp.70071 (PMC11922816; doi:10.1111/mpp.70071)
Supplement: Supplementary file 5 — Figure S5. [file MPP-26-e70071-s007.pdf]

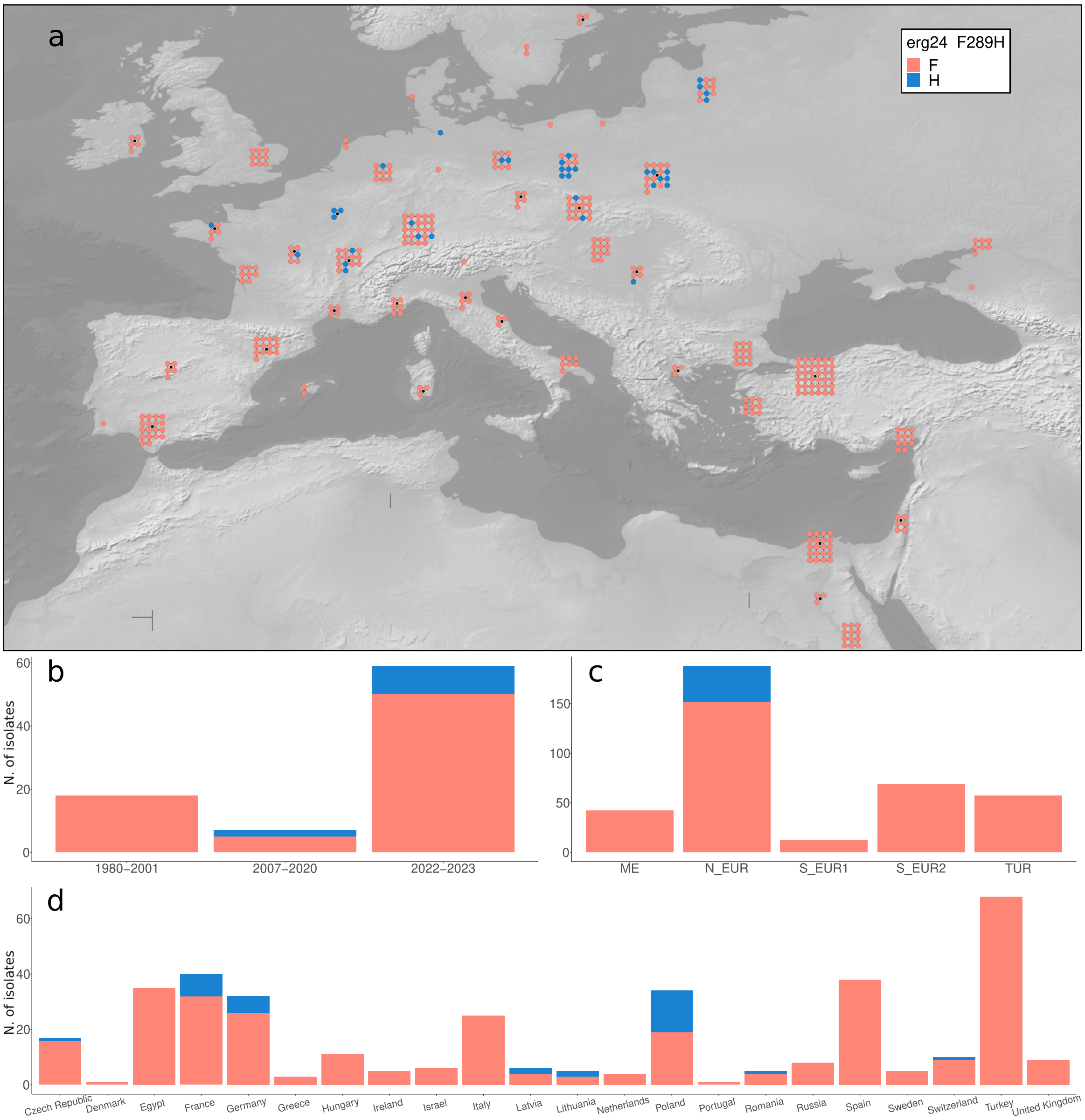

Figure S5. ***erg24* mutation F289H**
(a) Distribution of F289H. **(b)** Frequency of F289H by year of collection (*temporal* dataset). **(c)** Frequency of F289H by population. **(d)** Frequency of F289H by country of origin.
