# Supplementary material for: Genomic Surveillance and Molecular Evolution of Fungicide Resistance in European Populations of Wheat Powdery Mildew
Source: Mol Plant Pathol. 2025 Mar 19;26(3):e70071. doi: 10.1111/mpp.70071 (PMC11922816; doi:10.1111/mpp.70071)
Supplement: Supplementary file 6 — Figure S6. [file MPP-26-e70071-s009.pdf]

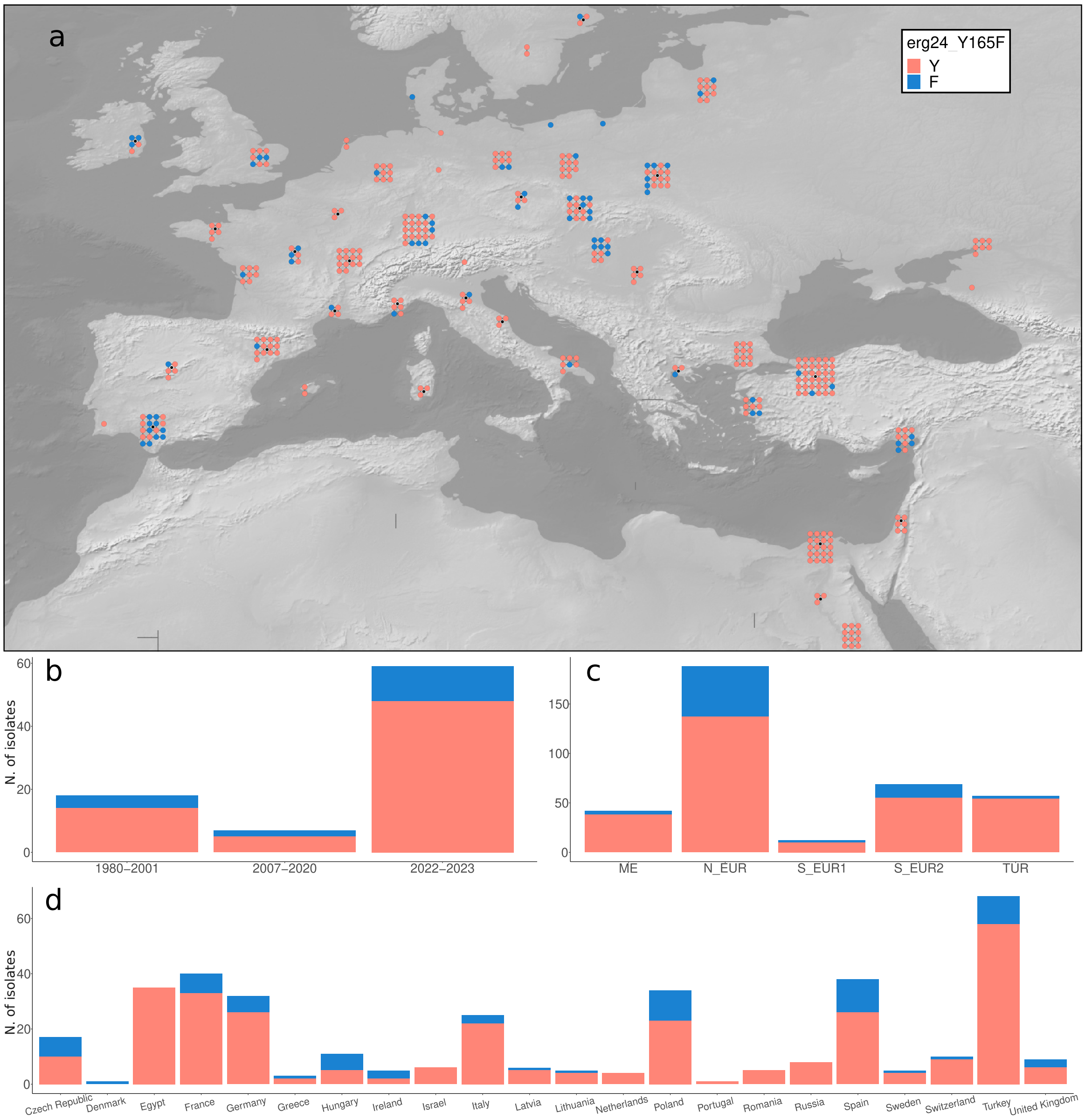

Figure S6. ***erg24*** **mutation Y165F**
(a) Distribution of Y165F. **(b)** Frequency of Y165F by year of collection (*temporal* dataset). **(c)** Frequency of Y165F by population. **(d)** Frequency of Y165F by country of origin.
