# Supplementary material for: Genomic Surveillance and Molecular Evolution of Fungicide Resistance in European Populations of Wheat Powdery Mildew
Source: Mol Plant Pathol. 2025 Mar 19;26(3):e70071. doi: 10.1111/mpp.70071 (PMC11922816; doi:10.1111/mpp.70071)
Supplement: Supplementary file 7 — Figure S7. [file MPP-26-e70071-s001.pdf]

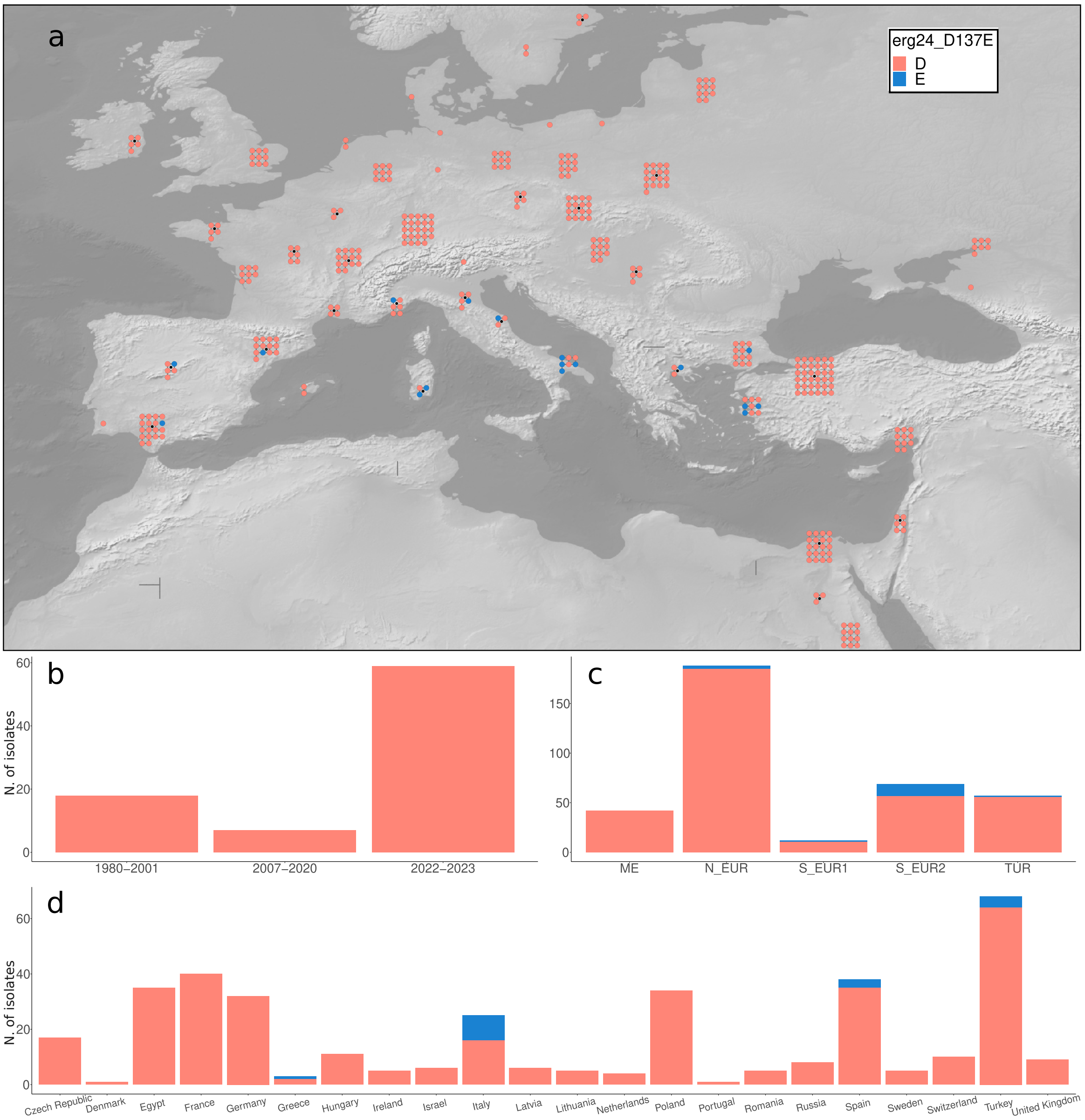

Figure S7. ***erg24* mutation D137E**
(a) Distribution of D137E. **(b)** Frequency of D137E by year of collection (*temporal* dataset). **(c)** Frequency of D137E by population. **(d)** Frequency of D137E by country of origin.
