# Supplementary material for: Genomic Surveillance and Molecular Evolution of Fungicide Resistance in European Populations of Wheat Powdery Mildew
Source: Mol Plant Pathol. 2025 Mar 19;26(3):e70071. doi: 10.1111/mpp.70071 (PMC11922816; doi:10.1111/mpp.70071)
Supplement: Supplementary file 8 — Figure S8. [file MPP-26-e70071-s011.pdf]

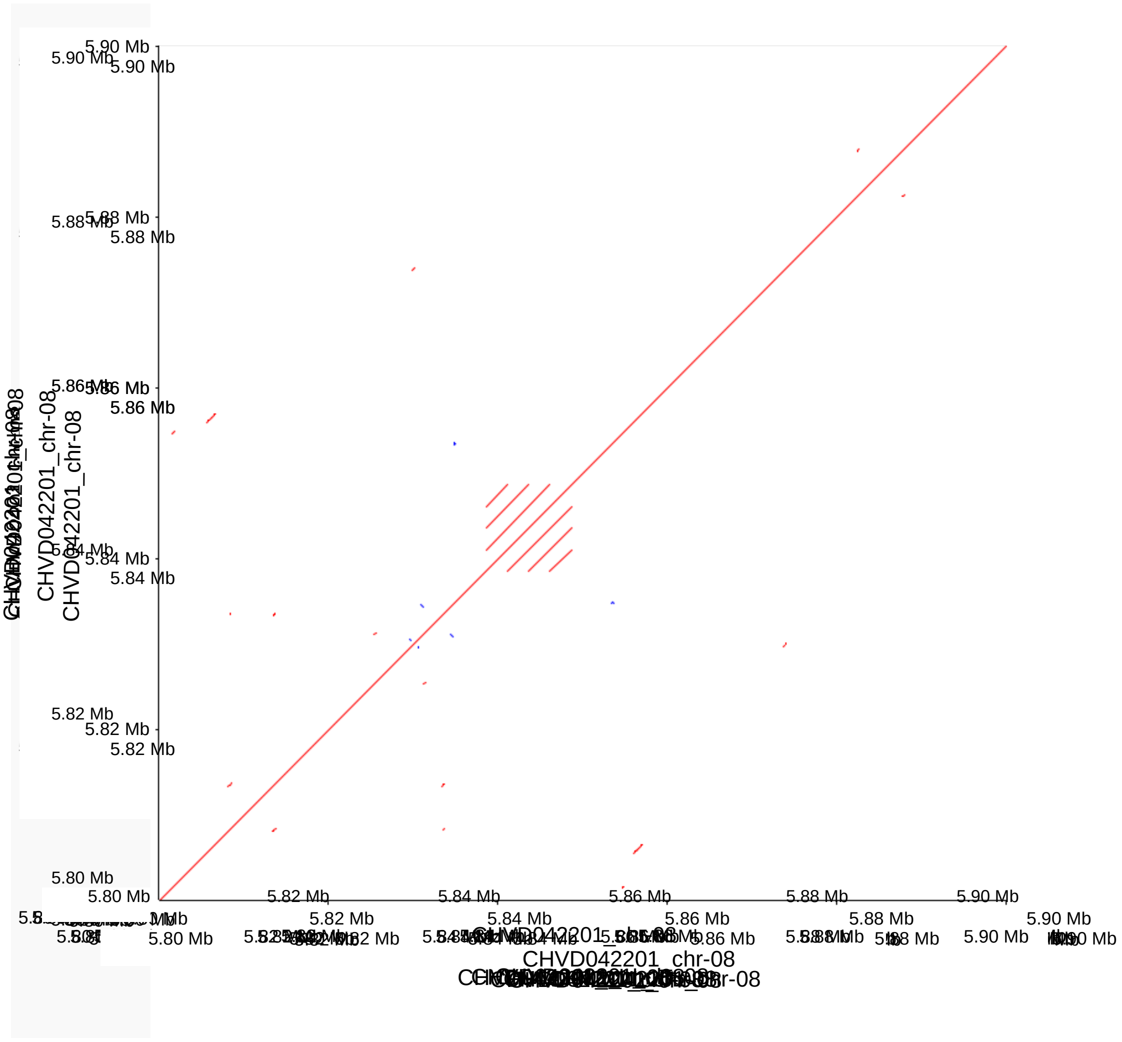

Figure S8. Dotplot of *cyp51* locus for isolate CHVD042201 Dotplot of 100Kb of chromosome 8 in correspondence of the *cyp51* locus. The same genomic fragment is plotted on the x and y axes. Four copies of *cyp51* are located in the tandem repeat.
