# Supplementary material for: Genomic Surveillance and Molecular Evolution of Fungicide Resistance in European Populations of Wheat Powdery Mildew
Source: Mol Plant Pathol. 2025 Mar 19;26(3):e70071. doi: 10.1111/mpp.70071 (PMC11922816; doi:10.1111/mpp.70071)
Supplement: Supplementary file 10 — Figure S10. [file MPP-26-e70071-s003.pdf]

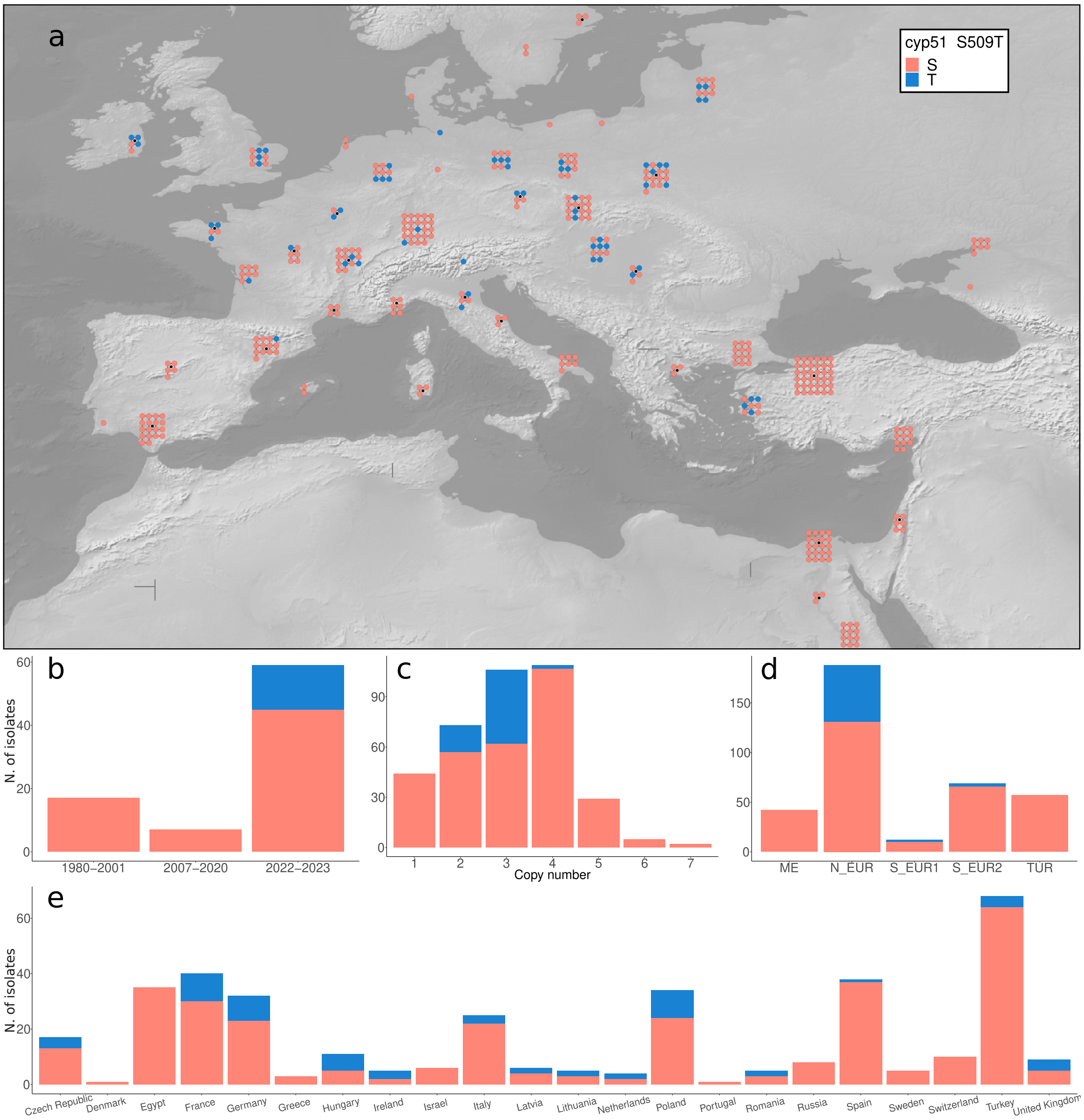

Figure S10. ***cyp51*** **mutation S509T**

(a) Distribution of S509T. **(b)** Frequency of S509T by year of collection (*temporal* dataset). **(c)** Frequency of S509T by population. **(d)** Frequency of S509T by country of origin.
