# Supplementary material for: Genomic Surveillance and Molecular Evolution of Fungicide Resistance in European Populations of Wheat Powdery Mildew
Source: Mol Plant Pathol. 2025 Mar 19;26(3):e70071. doi: 10.1111/mpp.70071 (PMC11922816; doi:10.1111/mpp.70071)
Supplement: Supplementary file 12 — Figure S12. [file MPP-26-e70071-s010.pdf]

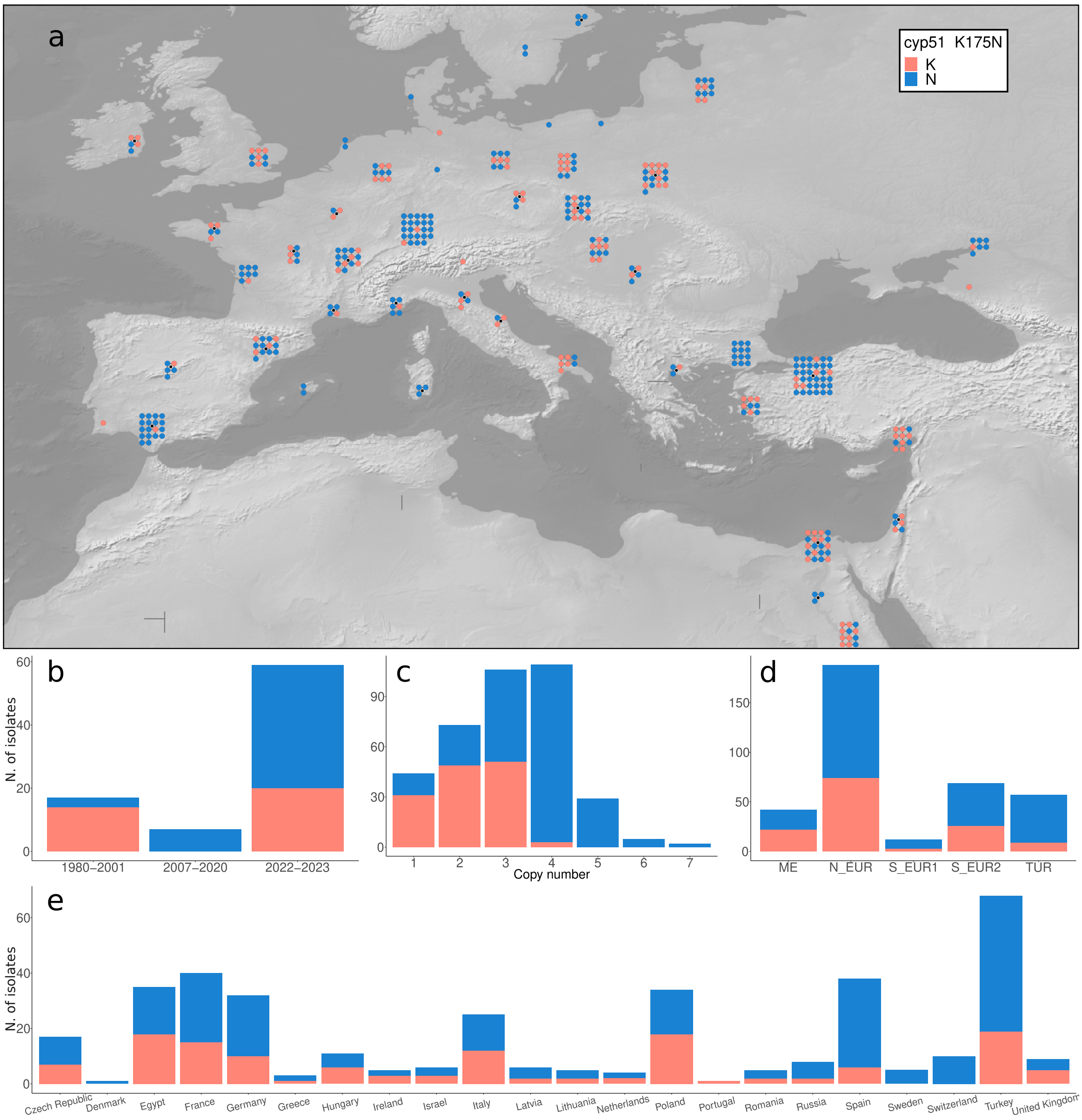

Figure S12. ***cyp51*** **mutation K175N**
(a) Distribution of K175N. **(b)** Frequency of K175N by year of collection (*temporal* dataset). **(c)** Frequency of K175N by population. **(d)** Frequency of K175N by country of origin.
