# Supplementary material for: Genomic Surveillance and Molecular Evolution of Fungicide Resistance in European Populations of Wheat Powdery Mildew
Source: Mol Plant Pathol. 2025 Mar 19;26(3):e70071. doi: 10.1111/mpp.70071 (PMC11922816; doi:10.1111/mpp.70071)
Supplement: Supplementary file 13 — Figure S13. [file MPP-26-e70071-s016.pdf]

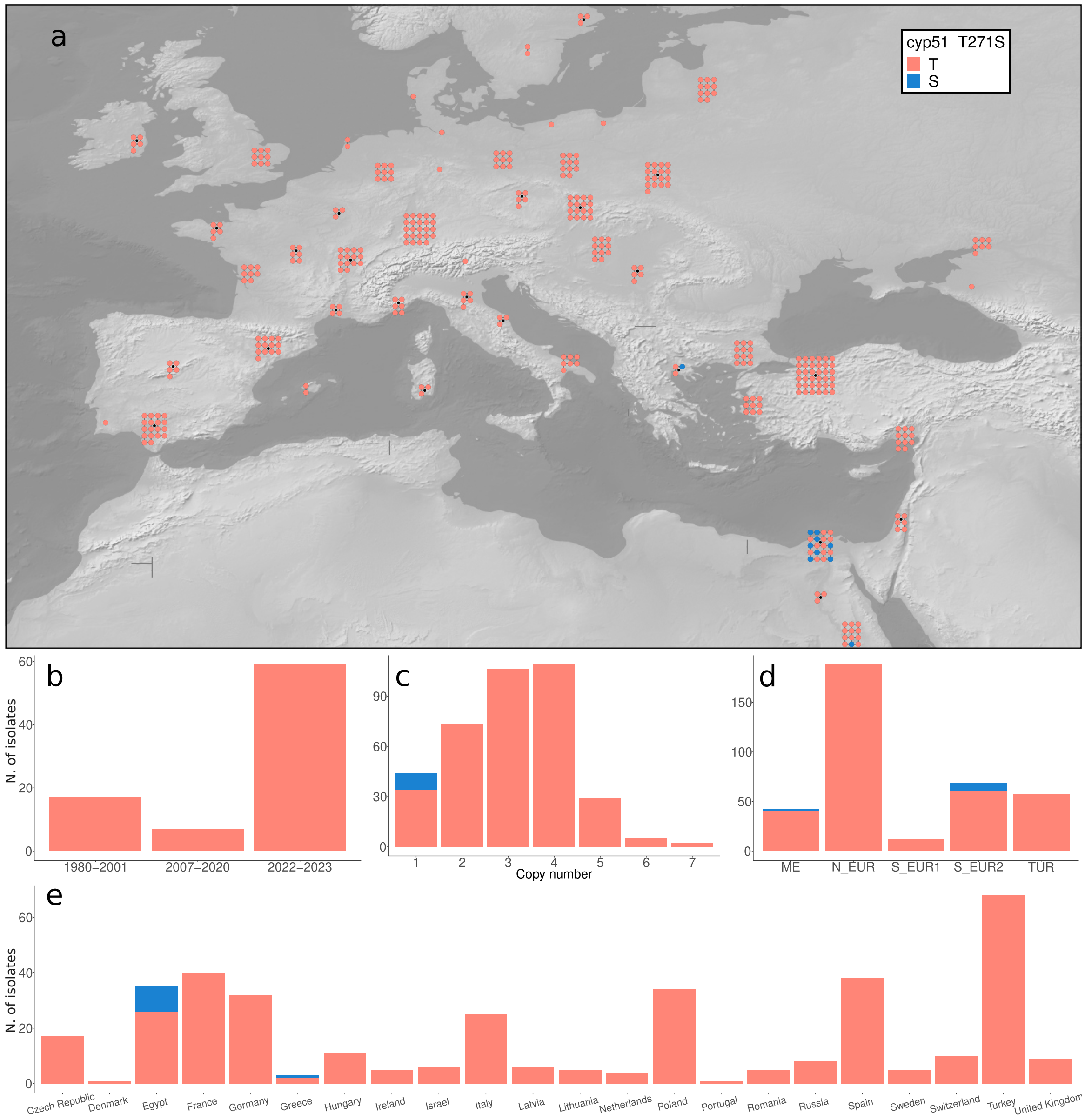

Figure S13. ***cyp51* mutation T271S**
(a) Distribution of T271S. **(b)** Frequency of T271S by year of collection (*temporal* dataset). **(c)** Frequency of T271S by population. **(d)** Frequency of T271S by country of origin.
