# Supplementary material for: Genomic Surveillance and Molecular Evolution of Fungicide Resistance in European Populations of Wheat Powdery Mildew
Source: Mol Plant Pathol. 2025 Mar 19;26(3):e70071. doi: 10.1111/mpp.70071 (PMC11922816; doi:10.1111/mpp.70071)
Supplement: Supplementary file 14 — Figure S14. [file MPP-26-e70071-s013.pdf]

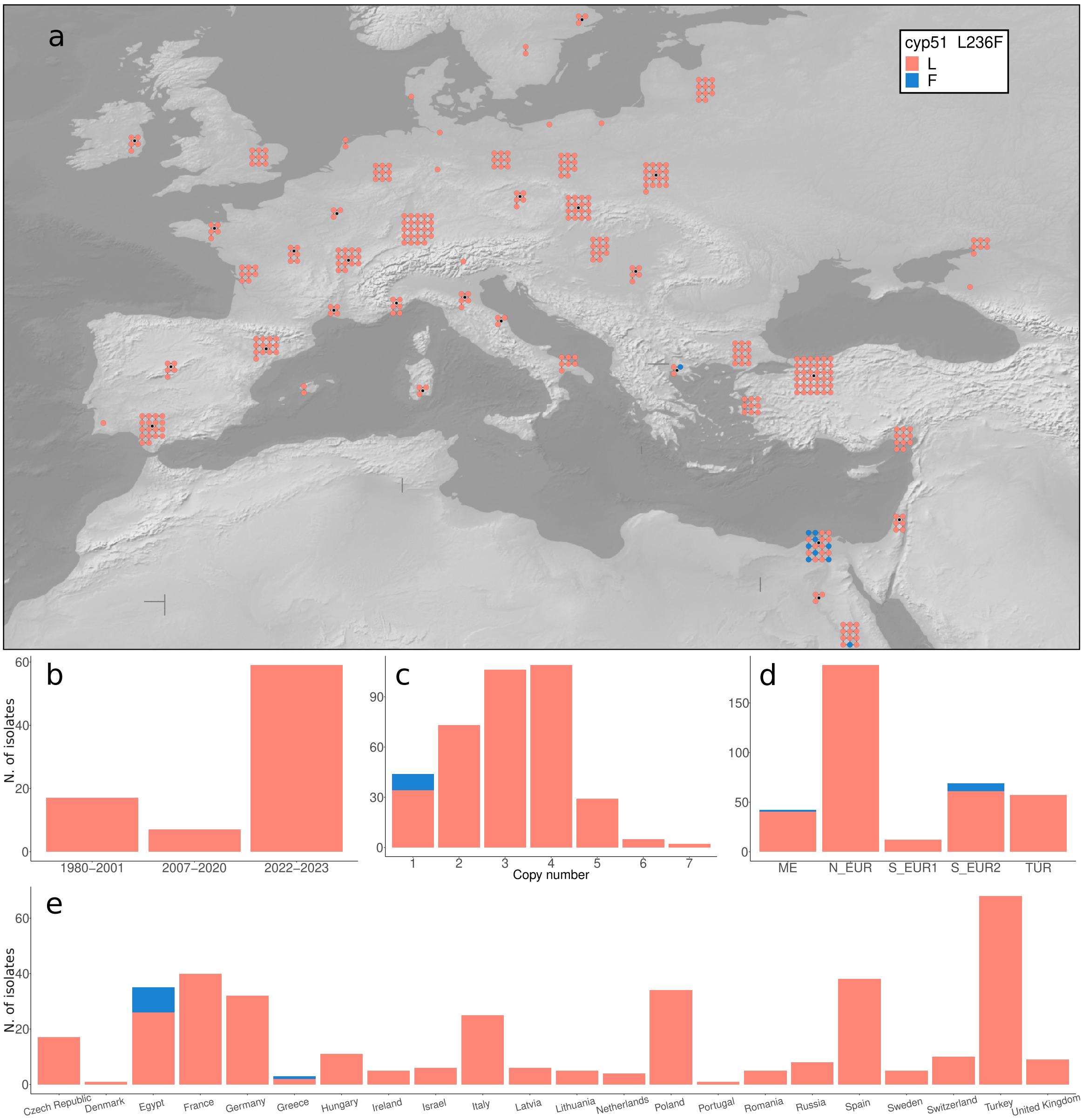

Figure S14. ***cyp51*** **mutation L236F**
(a) Distribution of L236F. **(b)** Frequency of L236F by year of collection (*temporal* dataset). **(c)** Frequency of L236F by population. **(d)** Frequency of L236F by country of origin.
