# Supplementary material for: Genomic Surveillance and Molecular Evolution of Fungicide Resistance in European Populations of Wheat Powdery Mildew
Source: Mol Plant Pathol. 2025 Mar 19;26(3):e70071. doi: 10.1111/mpp.70071 (PMC11922816; doi:10.1111/mpp.70071)
Supplement: Supplementary file 15 — Figure S15. [file MPP-26-e70071-s006.pdf]

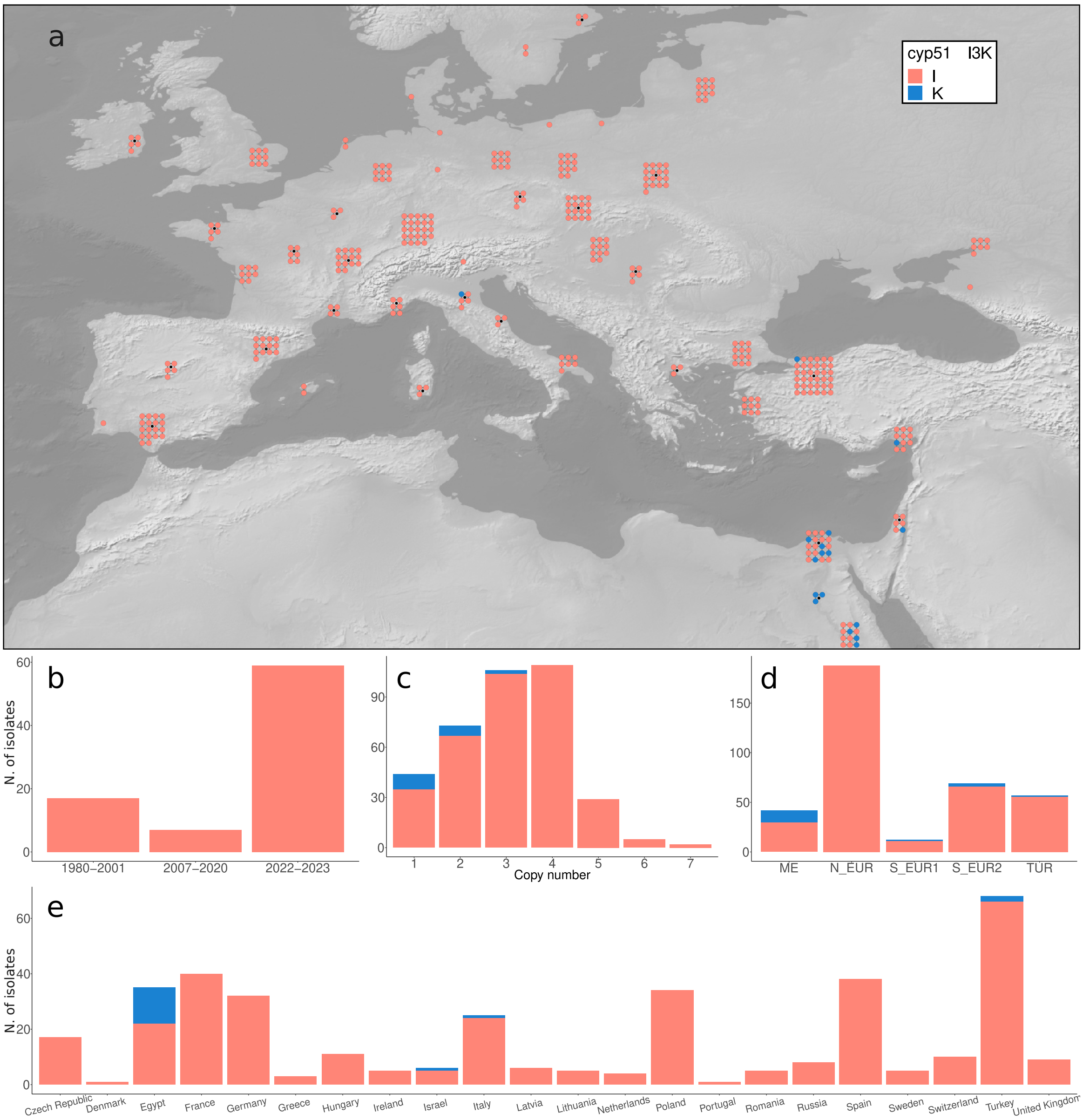

Figure S15. ***cyp51*** **mutation I3K**

(a) Distribution of I3K. **(b)** Frequency of I3K by year of collection (*temporal* dataset). **(c)** Frequency of I3K by population. **(d)** Frequency of I3K by country of origin.
