# Supplementary material for: Genomic Surveillance and Molecular Evolution of Fungicide Resistance in European Populations of Wheat Powdery Mildew
Source: Mol Plant Pathol. 2025 Mar 19;26(3):e70071. doi: 10.1111/mpp.70071 (PMC11922816; doi:10.1111/mpp.70071)
Supplement: Supplementary file 16 — Table S1. [file MPP-26-e70071-s008.pdf]

Table S1. All amino acid mutations in the eight target genes

The odds ratio and the p-values refer to the Fisher's exact test on the *temporal* dataset. For mutations not occurring in France, Switzerland and the UK the test could not be performed.

Gene target	Mutation	N. of isolates	Odds ratio	p-value
cytb	G143A	146	0.04769099	4.378 e-6
Btub	-	-	-	-
erg2	F29I	1	-	-
erg2	V59I	1	-	-
erg2	K89N	5	-	-
erg2	L115I	1	-	-
erg2	N177D	3	-	-
sdhB	A14T	1	-	-
sdhB	E36K	3	-	-
sdhB	A263S	1	-	-
sdhC	S9F	9	-	-
sdhC	S10L	1	-	-
sdhC	P29L	1	-	-
sdhC	F35I	2	-	-
sdhC	S38Y	3	-	-
sdhC	A130S	1	-	-
sdhD	T5I	1	-	-
sdhD	N22K	1	-	-
sdhD	H34N	1	-	-
sdhD	A90V	1	-	-
sdhD	A137V	1	-	-
erg24	D137E	17	-	-
erg24	Y165F	74	1.337203	0.732
erg24	H239N	1	-	-
erg24	F289H	36	0	0.1947
erg24	D291N	8	-	-
erg24	V295L	141	0.3555229	0.07493
erg24	F316F	1	-	-
erg24	L357F	1	-	-
cyp51	I3K	19	-	-
cyp51	S79T	211	0.1619583	0.002381
cyp51	Y136F	298	0.02111874	1.024e-05
cyp51	K175N	234	0.1133233	0.0006171
cyp51	L236F	14	-	-
cyp51	T271S	14	-	-
cyp51	E438K	1	-	-
cyp51	S509T	62	0	0.03079
